# Vaccination against β-Amyloid as a Strategy for the Prevention of Alzheimer’s Disease

**DOI:** 10.3390/biology9120425

**Published:** 2020-11-27

**Authors:** Francesca Mantile, Antonella Prisco

**Affiliations:** Institute of Genetics and Biophysics, CNR, 80131 Naples, Italy; Francesca.mantile@igb.cnr.it

**Keywords:** vaccine, Alzheimer’s disease, β-amyloid, AN1792

## Abstract

**Simple Summary:**

Immunization against β-amyloid has been explored as a vaccination strategy for Alzheimer’s disease for over 20 years. No vaccine has been licensed so far, and immunotherapy has come under considerable criticism following the negative results of several phase III clinical trials. In this narrative review, we illustrate the working hypothesis behind immunization against β-amyloid as a vaccination strategy for Alzheimer’s disease, and the outcome of the active immunization strategies that have been tested in humans. On the basis of the lessons learned from preclinical and clinical research, we discuss roadblocks and current perspectives in this challenging enterprise in translational immunology.

**Abstract:**

Vaccination relies on the phenomenon of immunity, a long-term change in the immunological response to subsequent encounters with the same pathogen that occurs after the recovery from some infectious diseases. However, vaccination is a strategy that can, in principle, be applied also to non-infectious diseases, such as cancer or neurodegenerative diseases, if an adaptive immune response can prevent the onset of the disease or modify its course. Immunization against β-amyloid has been explored as a vaccination strategy for Alzheimer’s disease for over 20 years. No vaccine has been licensed so far, and immunotherapy has come under considerable criticism following the negative results of several phase III clinical trials. In this narrative review, we illustrate the working hypothesis behind immunization against β-amyloid as a vaccination strategy for Alzheimer’s disease, and the outcome of the active immunization strategies that have been tested in humans. On the basis of the lessons learned from preclinical and clinical research, we discuss roadblocks and current perspectives in this challenging enterprise in translational immunology.

## 1. Introduction

Vaccination is an extremely effective public health intervention for infectious diseases and represents the most remarkable contribution of immunology to medicine [1]. 

Mechanistically, vaccination relies on the phenomenon of immunity, a long-term change in the immunological response to subsequent encounters with the same pathogen that occurs after the recovery from some infectious diseases. During the immune response, antigens induce the activation and differentiation of antigen-specific clones of B and T lymphocytes, that recognize different portions of the antigen, or epitopes. B cells (and antibodies that represent the soluble version of the B cell receptor for antigen) recognize exposed portions of the antigen, the B cell epitopes. Instead, the epitopes recognized by T cells consist of linear peptide sequences that are 8–12 aminoacid long in the case of cytotoxic T cells and 12–17 aminoacid long in the case of helper and regulatory T cells. Immunity relies on the differentiation of B cells into long-lived plasma cells, that ensure a persistent production of antibodies; moreover, B and T cells differentiate into memory cells that afford enhanced responses to subsequent encounters with the same antigen. 

Immunity is not an ‘all or nothing’ status. In sterilizing immunity, re-infection is completely prevented; in non-sterilizing immunity, the infection can occur but does not lead to disease, thanks to the mitigating effects of circulating antibodies against the pathogen and the enhanced speed, magnitude, and efficacy of the memory immune response. When a large fraction of a population is immune to an infectious pathogen, also members of the community that are not individually immune are protected from the disease, due to the reduced circulation of the pathogen. This phenomenon, termed herd immunity, only affects immunity to pathogens that are transmitted from one individual to another. Thus, vaccination against transmissible diseases consists of the induction of immunity, under conditions safer than the natural infection, and vaccination in general acts both at the level of the individual and the level of the community [2,3,4]. 

The last two decades have seen several attempts to harness the immune system’s power against Alzheimer’s disease (AD), by vaccinating against a peptide that has a central role in the pathogenesis, the β-amyloid peptide (Aβ). β-amyloid has been the target of several approaches to preventing and treating Alzheimer’s disease, including efforts to decrease the levels of Aβ monomers, oligomers, aggregates, and plaques using compounds that decrease production, antagonize aggregation, or increase brain clearance of Aβ [5]. Immunization against the β-amyloid peptide as a vaccination strategy for Alzheimer’s disease relies on the concept that antibodies against Aβ can interfere with its aggregation and accumulation, block its toxicity, or increase its catabolism, and on the hypothesis that these effects on brain Aβ may modify the course of the disease [6]. Obviously, the concept of herd immunity does not apply to vaccination against Alzheimer’s disease; in a vaccination for a non-infectious disease, only the vaccinated individuals that mount a response that meets the protective threshold are protected, and therefore the interindividual variability in the magnitude and quality of the immune response to vaccination is a particularly important issue. 

An important difference between vaccination against pathogens and vaccination against β-amyloid is the fact that the β-amyloid is a self-peptide. The failure to respond to self-antigens, defined immunological tolerance, is an essential feature of the immune system; autoimmunity is physiologically avoided by several mechanisms—e.g., clonal deletion of high affinity autoreactive lymphocytes in the thymus, editing of autoreactive B cell receptors, induced unresponsiveness in mature lymphocytes, and suppression by regulatory T cells. Vaccination against the β-amyloid peptide aims at inducing a controlled type of autoimmunity. In this context, one risk is that a vaccine may be unable to break tolerance, and therefore may not be immunogenic, while the opposite risk is that the vaccine may induce a damaging autoimmune reaction, and therefore may prove unsafe. Therefore, the immunogenicity and safety of anti-Aβ vaccines need to be carefully evaluated.

There is no licensed anti-β-amyloid vaccine or monoclonal antibody yet for Alzheimer’s disease. In clinical trials, immunotherapy against Aβ has been repeatedly unsuccessful. Some candidate vaccines have been abandoned following safety issues, despite displaying some efficacy, whereas other vaccines displayed a good safety profile but no efficacy; several vaccine candidates are currently in clinical trials. 

In this narrative review, we illustrate the working hypothesis behind anti-β-amyloid immunization, we summarize what has been learned so far from preclinical and clinical studies in terms of the safety and efficacy of this approach, and we discuss roadblocks and perspectives. We refer to the primary literature for the vaccination approaches, and to recent review articles for the neurological, neurochemical, and biochemical backgrounds.

## 2. Alzheimer’s Disease

Alzheimers’s disease (AD) is a progressive neurodegenerative disease. Symptoms include memory impairment and executive dysfunction interfering with daily life activities; as the disease progresses, patients gradually lose social and physical functions and independence [7]. 

Currently, no treatment is available that stops the progression of the disease; the disease causes a heavy personal toll on both patients and caregivers, and its management is financially costly. The prevalence of Alzheimer’s disease increases markedly with age, roughly doubling every 5 years after age 65. Thus, with the increase in life expectancy, the number of people living with Alzheimer’s, currently estimated to be around 40 million, is expected to grow; prevention or treatment of Alzheimer’s disease represents, therefore, a significant unmet medical need and an urgent issue in contemporary health care [8]. 

The large majority of Alzheimer’s disease cases (95%) are late-onset, that is, symptoms start after age 65, while about 5% of patients develop early-onset AD. Within early-onset cases, some cases are due to dominantly inherited mutations. These familial forms of the disease are particularly aggressive and tend to start at age 30–40 [9]. 

The risk for late-onset Alzheimer’s disease is partially driven by genetics. A large genome-wide association meta-analysis of clinically diagnosed late-onset AD, that confirmed 20 previous risk loci and identified five new loci, has shown that genetic variants affecting APP and Aβ processing are associated not only with early-onset dominantly inherited Alzheimer’s disease but also with late-onset AD [10]. A well-known risk allele for late-onset AD, ApoE4, decreases brain clearance of Aβ, leading to excess Aβ aggregation [11].

Alzheimer’s disease is defined histologically by the combined presence in the brain of extracellular senile plaques composed of Aβ and intracellular tangles of hyperphosphorylated tau. These neuropathologic findings distinguish AD from other diseases that can lead to dementia [9,12]. The neuropathologic changes in AD include accumulation of Aβ in the cerebral cortex in the form of plaques and in the blood vessel walls as cerebral amyloid angiopathy (CAA); phosphorylated tau in the form of tangles, neuropil threads, and plaque-associated dystrophic neurites; the activation of microglia and astrocytes; neuronal and synaptic dysfunction and loss, and cerebral atrophy [8]. 

Validated biomarkers exist that are proxies for the neuropathologic changes typical of Alzheimer’s disease. Aβ-related biomarkers, such as low Aβ42 in the cerebrospinal fluid (CSF) and positive amyloid-PET scans, precede other AD-related changes (increased CSF tau, decreased cerebral glucose metabolism, brain atrophy, clinical dementia) by years [13]. Both Aβ and tau biomarkers become abnormal years before the onset of cognitive symptoms, suggesting that the disease includes a long preclinical phase that can span up to two or three decades [12,14]. The preclinical phase could be an ideal time-window for preventative interventions to delay the onset of cognitive decline.

## 3. The β-amyloid Cascade Model of Alzheimer’s Disease Pathogenesis

**Figure 1:**
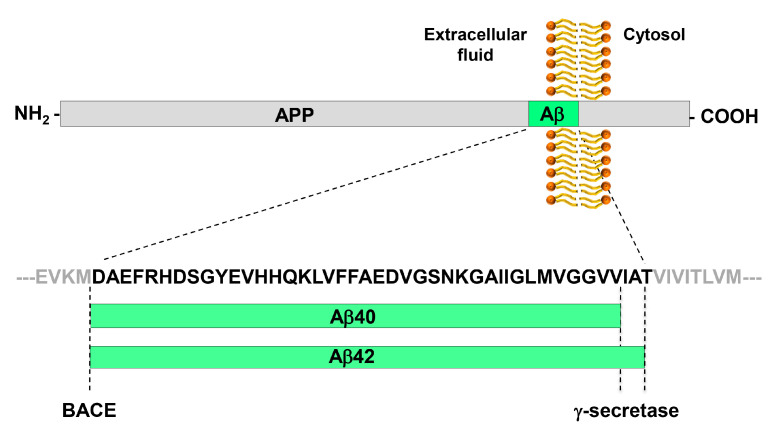
Generation of the Aβ peptide from the processing of APP by BACE and γ-secretase. APP is a type I trans-membrane glycoprotein. The β-secretase BACE has a single cleavage site on APP and generates the N-terminus of Aβ peptides. The γ-secretase has multiple cleavage sites on APP, which leads to the generation of Aβ peptides of variable length that differ for their C-terminus. The most abundant peptides are Aβ40 and Aβ42. Aβ42 is particularly prone to aggregation.

β-amyloid (Aβ) is a peptide, 38 to 43 amino acids long, that derives from the proteolytic processing of amyloid precursor protein (APP) by the γ-secretase; Aβ40 and Aβ42 are the most studied Aβ peptide species (Figure 1). The β-amyloid cascade model of Alzheimer’s disease pathogenesis consists in the hypothesis that an imbalance between production and clearance of Aβ42 and related Aβ peptides is a very early, often initiating factor in Alzheimer’s disease [13].

Strong evidence of the amyloid cascade model comes from the genetics of the ‘dominantly inherited forms of Alzheimer’s disease’ (DIAD). DIAD is caused by mutations that impact on the Aβ pathway, affecting either the amount of Aβ production or the ratio between the different forms of the peptide, in particular, by mutations within and immediately flanking the Aβ region of APP and by missense mutations in presenilin 1 and presenilin 2, the catalytic subunit of γ-secretase, that results in a relative increase in the production of Aβ42/43 peptides. In agreement with the concept that an excessive dose of Aβ causes Alzheimer’s, an enhanced gene dose of the precursor protein APP can cause the disease. In fact, APP is located on chromosome 21, and people with Down’s syndrome, who harbor three copies of APP, develop the typical neuropathology of Alzheimer’s disease at a young age [13]. 

**Figure 2:**
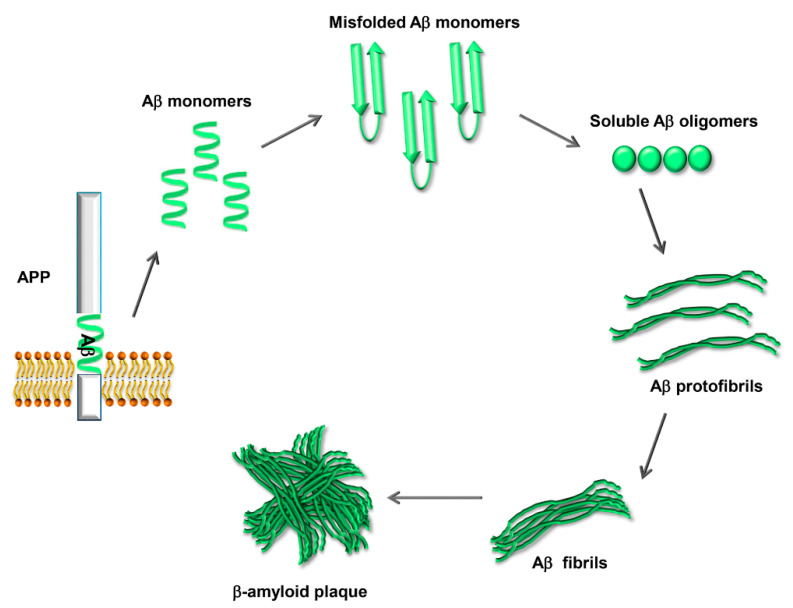
Process of Aβ aggregation and β-amyloid plaque formation. The Aβ peptide, once excised from APP, is prone to misfolding and self-aggregation. Misfolded Aβ monomers aggregate into small soluble oligomers. The oligomers interact to form protofibrils, which grow to form mature fibrils. Eventually the fibrils aggregate, forming the β-amyloid plaques.

The physiological functions of APP, the closely related APP-like proteins (APLPs), and their multiple processing products are still not well understood; there is evidence for a role in the development of the central nervous system, the formation and function of synapses, and neuroprotection following brain injury [15]. Aβ42, when excised from its precursor, is prone to undergo a conformational change that renders it able to self-aggregate into oligomers that can further assemble into fibrils and amyloid plaques (Figure 2). Amyloid fibrils are a structure that several proteins can adopt, characterized by a cross-β-sheet conformation in which β-strands run transversely to the main fiber axis and form an intermolecular network of hydrogen bonds [16].

Aβ plaque deposition is followed by neuritic and glial cytopathology in surrounding areas. A sequential association has been reported between brain β-amyloid accumulation, subsequent tau change, and resulting cognitive decline in individuals with dominant inherited AD [17]. While the amyloid plaques have been the target of many therapeutic approaches, the Aβ oligomers, which are diffusible and neurotoxic and exist in equilibrium with plaques, play the major role in neurodegeneration [18]. Aβ42 oligomers induce tau hyperphosphorylation and cause neuritic dystrophy in cultured neurons. In animal models, Aβ oligomers decrease synapse density, inhibit long-term potentiation, enhance long-term synaptic depression, and impair memory [19]. The most likely sequence of events leading to the disease comprises a neocortical Aβ accumulation, followed by a microglial inflammatory reaction to Aβ, neuritic dystrophy and spread of tau from the limbic system to the neocortex, and progressive tau accumulation and spread resulting in neurodegeneration [8,17]. Different therapeutic targets could be required for different stages of the disease process: Aβ for primary prevention, microglia for secondary prevention, and tau for established disease [8]. The β-amyloid peptide displays a highly complex self-assembly behavior, and it is challenging to define the aggregation process in terms of molecular events. The process is modeled as comprising primary and secondary pathways, that is, pathways that generate aggregates at a rate dependent on the concentration of monomers alone and independent of the concentration of existing fibrils (primary) and pathways that generate new aggregates at a rate dependent on the concentration of fibrils (secondary) [20]. Secondary pathways include mechanisms that depend only upon the concentration of fibrils, such as the fragmentation of fibrils, and mechanisms that depend on the concentration of both monomers and fibrils, such as secondary nucleation, whereby the surface of existing fibrils catalyzes the formation of oligomers from monomers. Secondary nucleation is a positive feedback loop in the aggregation process [20].

In agreement with this model of the aggregation process, oligomeric Aβ42 aggregates are particularly abundant around plaques; the neurodegeneration that is observed, in a gradient, around plaques is believed to derive mainly from the gradient of concentration of the diffusible, toxic oligomers [19,21]. Strategies to suppress the production of toxic oligomers need to consider both primary and secondary nucleation pathways of oligomer production. In this respect, while the oligomers and not the plaques are the direct culprit of the neurodegeneration, removing existing plaques makes sense as a step toward the reduction in the concentration of oligomers.

The early phases of the aggregation process reflect the fact that Aβ is a prion-like peptide, namely a peptide that adopts alternative conformations, which are self-propagating [22]. The self-propagating conformers, also known as ‘Aβ seeds’, can be quantified in cellular assays and in mouse models [22,23]. Aβ seeding potency is greatest early in the pathogenic cascade and diminishes as Aβ accumulates in the brain [22,24]. Interestingly, Aβ, as prions, can assemble into distinct strains of aggregates. Such strains may drive some of the phenotypic heterogeneity observed in Alzheimer’s disease [25]. 

## 4. Mechanisms of Action of Anti-β-Amyloid Antibodies 

**Figure 3:**
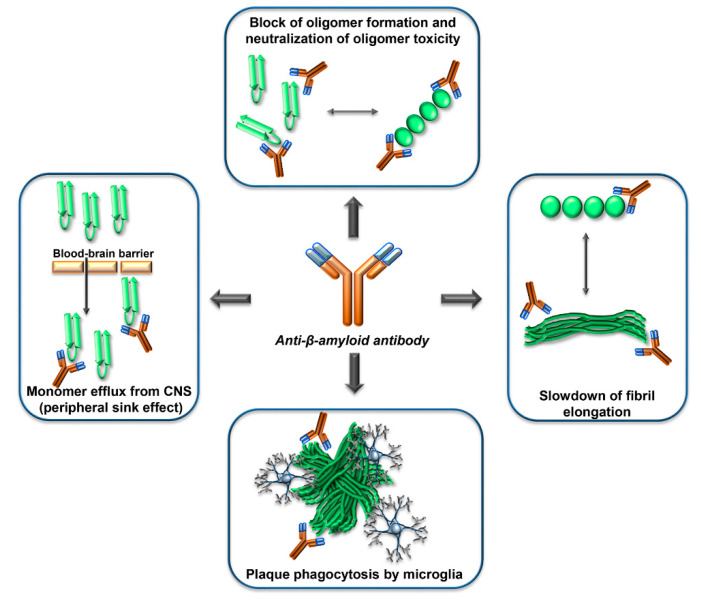
Mechanisms of action of anti-Aβ-antibodies. Anti-Aβ-antibodies can interfere with the β-amyloid cascade at several levels, by interacting with the Aβ monomers, the Aβ oligomers, the Aβ-fibrils, or the Aβ-plaques. The epitope specificity and concentration of antibody required for the different mechanisms are probably different. In human clinical trials, evidence of phagocytosis of plaques by microglia has been reported. The ‘peripheral sink effect’ is a hypothesis that has been disproven.

Many studies in vitro, in cell cultures and animal models, have shown that antibodies against β-amyloid can exert potentially useful effects to counteract β-amyloid dependent neurodegeneration, such as interfering with β-amyloid aggregation, blocking β-amyloid toxicity, or reducing the amount of β-amyloid in the brain (Figure 3). 

The entry of antibodies in the brain is restricted by the blood–brain barrier [26]; the concentration of an antibody in the CSF is 0.1 to 0.2% of the concentration in blood plasma [27,28]. Peripherally administered antibodies against the β-amyloid peptide can enter the central nervous system and reduce amyloid load; this was first documented in mouse models of Alzheimer’s Disease [29], and more recently also observed in clinical trials of passive immunization [30].

Different mechanisms of action of anti-β amyloid antibodies have been hypothesized or documented, including allosteric effects, the induction of plaque phagocytosis by microglia, the promotion of efflux of Aβ from the CNS to the circulation, the neutralization of β-amyloid toxicity (Figure 3). It is reasonable to expect that different mechanisms can become relevant depending on the stage of the amyloid deposition process, and on the concentration, isotype, and epitope specificity of the antibodies. 

Amyloid deposition appears to follow a sigmoidal trajectory over time [31]. The time window where the slope of the amyloid load versus time curve is greatest represents a potential therapeutic window for secondary preventive interventions [31]. On the other hand, therapeutic interventions designed to reduce the rate of new amyloid deposition, rather than removing previously deposited amyloid, may be less effective in patients who have already reached plateau levels of amyloid deposition [31].

In vitro, anti-Aβ monoclonal antibodies can prevent Aβ monomers from forming fibrillar aggregates, and can convert fibrillar aggregates into an amorphous state [32]. Interestingly, the efficacy of these mechanisms depends on the concentration of antibody. At low concentrations of AMY-33, a monoclonal antibody raised against β-amyloid fragment 1–28, only amorphous aggregates are formed; increasing the concentration of antibody to equimolar antigen/antibody ratios maintained the solubility of β-amyloid [32]. The solubilization of already formed aggregates also required an equimolar ratio of antigen/antibody ratio, and prevented the neurotoxicity of β-amyloid [33]. 

Some epitopes of Aβ are preferentially available within plaques, whereas other epitopes are only available on the soluble peptide. Therefore, the epitope specificity of an antibody affects its binding affinity against different β-amyloid species. For epitopes exposed both in the monomer and in the aggregate forms, the presence of two antigen-biding sites generates avidity effects for aggregates. Therefore, the preferential binding of an antibody to the aggregate forms does not necessarily imply the recognition of a conformational epitope only present in the aggregate species. For instance, the sera of mice immunized with a multimeric protein that displays the Aβ(1–11) peptide [34,35], recognize the synthetic Aβ(1–11) peptide in ELISA assay, but display a marked preference for oligomeric and fibrillar species of β-amyloid in dot blot assays [36]. 

It has been hypothesized that soluble β-amyloid levels in the brain and the peripheral blood are in equilibrium so that blocking or degrading β-amyloid in blood should increase its efflux from the brain, and reduce brain amyloid. This hypothesis, known as the peripheral sink hypothesis [37], has been refuted. In various experimental systems, treatments that substantially decreased peripheral Aβ levels failed to affect brain and cerebrospinal fluid levels of Aβ; these findings suggest a lack of a significant peripheral sink effect through which brain amyloid burdens can be therapeutically reduced [38,39,40]. In clinical trials, a monoclonal antibody that binds soluble Aβ in blood, solanezumab, did not affect brain levels of amyloid [41]. When Aβ in blood is bound to solanezumab, the half-life of the Aβ-antibody complex is much longer than the half-life of free Aβ; therefore, treatment with solanuzemab leads to an increase in of the concentration of Aβ in blood, but the increase is not due to an efflux of Aβ from the brain. 

While the fragment of the antibody that binds the epitope determines the specificity of the antibody, the constant region of the antibody, or Fc, is responsible for the capacity of an antibody to activate the complement cascade or to bind the Fc receptors that are expressed on a wide variety of cell types of the immune system, including microglia in the central nervous system; different antibody isotypes differ in their affinity for Fc receptors and ability to activate complement. The antibody isotype, therefore, affects Fc- or complement-mediated phagocytosis of plaques by microglial cells. An analysis of the epitope and isotype specificity of antibodies against β-amyloid able to protect against Alzheimer’s disease concluded that epitopes within the N terminus of Aβ are important for plaque clearance and neuronal protection via an Fc-mediated mechanism, and that IgG2a antibodies against Aβ are more efficient than IgG1 or IgG2b antibodies in reducing neuropathology [42]. 

## 5. Effect of Anti-β-amyloid Vaccination in Mouse Models of β-Amyloid Deposition

Genetically engineered mouse models have been instrumental to Alzheimer’s disease research and preclinical drug development. Transgenic mouse strains that overexpress mutant human APP linked to familial AD progressively develop many of AD’s pathological hallmarks—including senile plaques, synaptic loss, astrocytosis, and microgliosis—and have been largely used as preclinical research models. Recently, new mouse models have been generated that contain humanized sequences and clinical mutations in the endogenous mouse APP gene [43].

The first report of a disease-modifying effect of immunization against β-amyloid was published over 20 years ago. A transgenic mouse model of Alzheimer’s disease, the PDAPP mouse, was immunized with Aβ42, either before the onset of neuropathology, or at an older age, when amyloid-β deposition and neuropathology were well established. Immunization of the young animals prevented the development of β-amyloid-plaque formation, neuritic dystrophy, and astrogliosis, and treatment of the older animals markedly reduced the neuropathology [44]. 

A study performed in a different transgenic mouse model of Alzheimer’s disease, the TgCRND8 mouse, reported that Aβ immunization reduced both the deposition of cerebral fibrillar Aβ and cognitive dysfunction without, however, altering total levels of Aβ in the brain. The authors suggested that either a 50% reduction in dense-cored Aβ plaques was sufficient to affect cognition, or vaccination may modulate the activity/abundance of a small subpopulation of especially toxic Aβ species [45].

In yet another transgenic mouse strain, the Tg 2576 APP transgenic mice, and in a double mutant including both the APP and a PSEN mutation, vaccination against Aβ afforded protection from memory impairment, in the presence of reduced—but still substantial—Aβ deposits. The authors hypothesized that the antibodies could neutralize Aβ in some restricted compartment or deplete a non-deposited form of Aβ (for example, a soluble form) responsible for the memory loss [46]. 

The perspective of injecting the Aβ1–42 peptide in healthy, young humans to vaccinate them against Alzheimer’s raises in principle a safety concern over the possibility that the injected peptide itself, being able to promote oligomerization and fibrillogenesis, may start the pathogenic cascade in some healthy vaccinees, causing an enhanced probability of incurring the disease decades later. This scenario still cannot be formally excluded. Therefore, it is important to bear in mind that, ideally, the molecules used to immunize against β-amyloid should not be able to initiate the amyloid cascade. So far, all human immunizations have been performed in individuals that already had aggregated β-amyloid in their body. Importantly, in mice, it is unnecessary to immunize with the entire, pre-aggregated Aβ1–42 to observe an effect on brain amyloid. Immunization of transgenic APP mice with a soluble nonamyloidogenic, nontoxic Aβ homologous peptide, consisting of the first 30 amino acid residues of Aβ with six additional lysine residues at the N-terminus, reduced cortical and hippocampal brain amyloid burden and brain levels of soluble Aβ1–42, and reduced neuroinflammation [47]. Later in this review, in the paragraph on second generation vaccines, we illustrate immunization strategies that avoid the use of full length β-amyloid.

## 6. Anti-β-Amyloid Vaccination in Humans with Vaccine AN1792

The Aβ1–42 peptide, in a pre-aggregated form, has been tested as a vaccine in humans under the name AN1792. AN1792 is the anti-β amyloid active immunization attempt on which more data has been published, with the longest follow-up. Therefore, we will review the results of the human immunizations here in detail.

AN1792 was formulated with QS21, an adjuvant able to enhance antibody responses and to favor a Th1 polarization of the T cell response [48]. Activated T cells can differentiate into subsets characterized by the productions of different sets of cytokines and different functions. The Th1 subset is characterized by its ability to secrete IFN-γ, a pro-inflammatory cytokine.

In the phase I trial, patients with mild to moderate AD received injections of AN1792 + QS-21 on day 0 and at weeks 4, 12, and 24. Patients could receive up to four additional injections of a polysorbate 80 modified formulation at weeks 36, 48, 60, and 72 [49]. During the period of the first four injections, 23.4% of AN1792-treated patients had an anti-AN1792 antibody titer of ≥1:1000. This increased to 58.8% after additional injections with the polysorbate 80 modified formulation. Disability Assessment for Dementia scores showed less decline among active compared with control patients at week 84 [49].

Thus, the phase I trial of AN1792 demonstrated that AN1792 + QS-21 was able to elicit an antibody response to Aβ42 [49], and also indicated some efficacy on the progression of the disease. The safety of the immunization was considered acceptable. One patient developed meningoencephalitis, that was diagnosed after death, and at the time was not considered to be related to the study treatment. AN1792 therefore proceeded to phase II trials. However, all clinical trials of AN1792 were interrupted when, in the phase II trial, meningoencephalitis occurred in 6% (18/300) of immunized patients [50]. 

In the phase II trial, the dosing protocol included intra muscular injections at baseline and at months 1, 3, 6, 9, and 12. As patients started to develop the meningoencephalitis reactions, however, dosing was discontinued, after only one to three injections. The predefined serum antibody response (anti-AN1792 IgG titer ≥ 1:2200) was achieved in 59 out of 300 patients (19.7%) [51]. Of the 18 patients that developed signs of meningoencephalitis, one had received one dose, 16 had received two doses, and one had received three doses before the symptoms of meningoencephalitis occurred [51].

### 6.1. Short Term Effects of AN1792 Immunization—One Year Follow-Up

In a Zurich cohort of 30 patients who had participated to the multicenter phase IIa trial the generation of antibodies against β-amyloid plaques correlated with clinical stabilization. Over the observation period of one year, patients with strong increases in anti-plaque antibodies remained clinically and cognitively stable, whereas the cognition of patients that had not generated the antibodies worsened [52]. The anti-plaques antibodies measured by tissue amyloid plaque immunoreactivity (TAPIR) [53] predicted outcome, whereas the anti β-amyloid titer measured by ELISA did not predict outcome [52]. This observation suggests that the antibody response to AN1792 may be qualitatively different in different individuals, and that the quality of the antibody response can affect the outcome.

In the general analysis of all the data from the phase IIa trial [51], although no significant differences were found between antibody responders and placebo groups for the various scales of cognitive assessment used, the Neuropsychological Test Battery (NTB) revealed differences favoring antibody responders. Greater improvements from baseline were associated with higher IgG antibody titers [51]. Moreover, tau in the CSF was decreased in antibody responders vs. placebo subjects [51].

### 6.2. Long Term Effects of AN1792 Immunization

The long term effects of AN1792 immunization was first reported on a small subset patients from the phase I trial, who consented to the clinical follow-up and post-mortem neuropathological examination [54]. In the immunized participants the mean Aβ load was lower than in unimmunized controls matched for age at death [54]. The mean antibody response attained during the treatment study period appeared to affect the degree of plaque removal [54]. Although immunization with Aβ42 resulted in clearance of amyloid plaques in patients with Alzheimer’s disease, this clearance did not prevent progressive neurodegeneration; also patients with the highest mean antibodies to Aβ and virtually complete plaque removal reached severe end stage dementia [54], implying that progressive neurodegeneration can occur in Alzheimer’s disease despite removal of plaques.

A larger follow-up study was conducted to assess the long-term outcomes 4.6 years after immunization with AN1792, to determine if benefits might accrue over time in patients from the phase IIa who had developed the pre-defined antibody titers (above 1:2200); patients originally identified as antibody responders were compared with placebo-treated patients [55]. Antibody responders retained low but persistent anti-AN1792 antibody titers after approximately 4.6 years. Compared with placebo-treated patients, antibody responders demonstrated significantly less impairment in activities of daily living and significantly less dependence on caregivers, and tended to perform better on the memory component of the Neuropsychological Test Battery [55].

A 15-year post-mortem neuropathological follow-up of patients from the phase I trial of AN1792 has investigated the relationships between the topographical distribution of amyloid-β removal from the cerebral cortex and tau pathology, cerebrovascular territories, anti-AN1792 antibody titers, and late cognitive status [56]. Fourteen of 16 (88%) Alzheimer’s patients who had received the active agent had evidence of plaque removal. Two Alzheimer’s patients who died 14 years after immunization had only very sparse or no detectable plaques in all regions examined. Despite modification of Alzheimer’s pathology, most patients had progressed to severe dementia, notably including those with very extensive plaque removal, possibly due to continued tau propagation [56]. Nevertheless, the study demonstrated that patients with Alzheimer’s disease actively immunized against amyloid-β can remain virtually plaque-free for 14 years. The extent of plaque removal was related to the anti-AN1792 antibody response [56].

### 6.3. Specificity of Antibodies Induced by AN1792

The immune sera from patients immunized with AN1792 specifically recognized β-amyloid plaques and diffuse Aβ deposits, as well as vascular amyloid in subarachnoidal and perforating brain vessels [53]. The immune sera did not cross-react with either denatured or native full-length APP [53]. Hock et al. reported no immunoreactivity of the sera against soluble Aβ42, dimers and trimers [53]. Epitope mapping with 10mer peptides mapped the antibody response, in 42 subjects, to the first 10 amino acids of Aβ42 (DAEFRHDSGY); the exposed N terminal amino acid appeared part of the epitope, as peptides extending N terminally to include APP sequence were poorly recognized by sera, confirming that the antibody response induced by Aβ42 does not cross-react with APP [57]. Pre-absorption with the amino terminal peptide Aβ(1–8) removed plaque-binding activity of sera, suggesting that the antibodies induced by AN1792 recognize a linear epitope, and not a specific conformation or multiple of Aβ unique to amyloid plaques [57]. At a difference with the data from Hock et al. [53], the data from Lee at al. [57] indicate that immune sera with high titer from patients immunized with AN1792 recognize also monomeric Aβ42, and that the antibodies generated by immunization with the pre-aggregated synthetic peptide recognize a linear epitope, not a conformational epitope [57].

### 6.4. Meningoencephalitis Reaction Induced by AN1792

The meningoencephalitis reactions that were observed during the AN1792 phase II experimentation were clearly associated to the treatment; none of the participants that had received placebo developed meningoencephalitis. Meningoencephalitis occurred without clear relation to serum anti-Aβ42 antibody titers [58]; five of the 18 patients that had experienced meningoencephalitis did not show the predetermined anti-β-amyloid antibody response, that is a titer higher than 1:2200, and one never developed a measurable antibody response [51].

The first neuropathological case report of an AD patient immunized against β-amyloid who had developed meningoencephalitis, proposed that the vaccination had caused Aβ plaques’ clearance [59].The patient had participated to a phase I trial of immunogenicity. After the first dose and subsequent doses at 4, 12, and 24 weeks, the woman had suffered no apparent adverse effects. Thirty-six weeks after the first injection, the woman had received a fifth injection with a reformulated preparation containing polysorbate-80. Six weeks later, she had become unwell, deteriorating such that cognitive tests could not be performed; her conditions remained relatively unchanged until she died, one year after the last injection. Comparison with unimmunized cases of AD revealed that, in the immunized patient, there were extensive areas of neocortex with very few Aβ plaques. These areas contained tangles, neuropil threads and cerebral amyloid angiopathy similar to unimmunized AD, but lacked plaque-associated dystrophic neurites and astrocyte clusters. In some of these plaque-free areas, Aβ-immunoreactivity was associated with microglia, the resident macrophages of the central nervous system; during development and homeostasis, microglial phagocytosis is essential for the refinement of synapses, and for the removal of apoptotic cells and debris [60].

All these findings suggested that the immune response generated against the peptide had elicited clearance of Aβ plaques, and that microglia cells had phagocytosed plaques. In the analysis, T-lymphocyte meningoencephalitis and infiltration of cerebral white matter by macrophages were also observed, and identified as possible correlates of the adverse reaction [59]. In this case report, extensive and persistent plaque removal clearly had afforded no clinical benefit, whereas the effect of the adverse reaction had been long-lasting. A neuropathological analysis of the brain of another trial participant who had received two intramuscular injections of AN1792 with adjuvant QS-21, separated by one month, and had experienced meningoencephalitis 6 months later, also reported a reduction of β-amyloid and T cell infiltration, and also multiple small hemorrages [61]. The absence of plaques was also reported in a patient immunized with AN-1792 who did not experience meningoencephalitis [62]. In this case, there were no amyloid plaques in the frontal cortex and abundant Aβ-immunoreactive macrophages, but tangles and amyloid angiopathy were present. The white matter appeared normal and minimal lymphocytic infiltration in the leptomeninges was observed [62]. This case demonstrated that Aβ immunization can affect brain amyloid in the absence of overt meningoencephalitis and leukoencephalopathy. 

Overall, the meningoencephalitis reactions were attributed to the T cell response. In the phase IIa study, in which meningoencephalitis reactions were more frequent than in the phase I study, T-cell responses to Aβ were Th1-biased, and it was hypothesized that the meningoencephalitis might be associated with Th1 CD4 T cells, which are known to be pro-inflammatory, or with CD8, cytotoxic T cells. The epitope of antibodies were similar in the phase I study and the phase II [63]. Polysorbate 80, used in the phase II study, was considered a possible explanation for the different polarization of the T cell response in the different studies [63].

In the Zurich cohort, two patients with aseptic meningoencephalitis and who generated antibodies against β-amyloid experienced a transient worsening of cognition, but then recovered and remained cognitively stable one year after the immunizations, suggesting that the beneficial effects of antibodies against β-amyloid on cognitive functions are maintained even after transient episodes of meningoencephalitis [52]. 

### 6.5. Effect of AN1792 Immunization on Brain Volume and Brain Vasculature

In Alzheimer’s disease, progressive neurodegeneration involves brain atrophy over time, which is detectable in vivo by magnetic resonance imaging (MRI), and can be used as a marker of disease progression. Quite surprisingly, the MRI findings of the Phase IIa trial revealed that, one year after the start of immunization, antibody responders had greater brain volume decrease, and greater ventricular enlargement than placebo patients [64]. These increased losses in brain volume were not reflected in worsening cognitive performance [64]. The decrease in brain volume was transient. Placebo-treated patients and antibody responders did not demonstrate significant differences in loss of brain volume approximately 3.6 years from the end of the phase IIa study [55]. These observations revealed that amyloid removal and associated cerebral fluid shifts can result in macroscopic effects on brain volume, not due to neuronal degeneration [64].

Since antibodies can dissolve aggregated Aβ, an important issue about anti-Aβ immunotherapy is the fate of the solubilized Aβ. In aged APP-transgenic mice treated with passive immunotherapy against Aβ, as solubilized Aβ drains via the vascular pathway, vascular amyloid and microhemorrhages increase [65]. In mice treated with passive immunotherapy against Aβ, clearance of Aβ plaques and clearance of Aβ from vessels follows distinct kinetics [66]. It has been hypothesized that solubilized Aβ drains via the perivascular pathway, causing a transient increase in the severity of cerebral amyloid angiopathy [67]. This hypothesis is supported by the neuropathological examination on nine patients who died between four months and five years after their first immunization with AN1792. Compared with non-immunized Alzheimer’s patients, immunized patients had more blood vessels containing Aβ42 in the cerebral cortex and the leptomeninges, a significantly higher level of cerebrovascular Aβ40, and a higher density of cortical microhemorrhages and microvascular lesions. Two of the longest survivors, who had lived four to five years after first immunization, had virtually complete absence of both plaques and cerebral amyloid angiopathy, raising the possibility that the increase in the severity of CAA is transient, and that immunotherapy at later timepoints clears Aβ also from the cerebral vasculature. A similar observation has been reported in the context of passive immunization; a massive vascular amyloid burden has also been reported in a patient treated with the monoclonal anti-β-amyloid antibody solanezumab [68]. It remains to be established if it represented solubilized β-amyloid mobilized to the brain vasculature.

In subsequent clinical trials of anti-β-amyloid immunotherapy, the effects on brain vasculature have been monitored in vivo, by magnetic resonance imaging (MRI). The side effects of anti-β-amyloid treatment that can be detected by MRI have been named amyloid-related imaging abnormalities (ARIA). In particular, two types of ARIA have been defined: ARIA-E, or vasogenic edema; and ARIA-H, indicating microhemorrhages and hemosiderosis [69].

ARIA-E and ARIA-H are induced by anti-Aβ antibodies [70,71], and therefore can occur both in passive and in active anti-Aβ immunotherapy. In the prospect of developing a safe immunotherapy, it is important to identify the patients more at risk of incurring serious events, and to understand how to best manage these events. The APOE 4 allele, in a study with anti-Aβ monoclonal Bapineuzumab, appeared to be a risk factor for ARIA [71].

## 7. Anti-β-Amyloid Vaccination in Humans with Second Generation Vaccines

Despite the serious adverse events, the results of the AN1792 clinical trials encouraged further research into active anti-Aβ immunotherapy. Since the meningoencephalitis was attributed to the T cell response, mapped to the central part of the β-amyloid peptide, and the effects on disease progression were attributed to the antibody response, mapped to the N-terminus of Aβ, second generation vaccines mainly used the strategy of directing the immune response to the N-terminal B cell epitope, without inducing a concomitant T cell response to β-amyloid. 

The active immunogens that have entered clinical trials are shown in Table 1. Four anti-Aβ vaccines are currently being tested in phase II trials: CAD106, ACI-24, UB-311, and ABVac40.

The T cell response is important for the generation of high affinity IgG; several second generation anti-Aβ vaccines include exogenous T cell epitopes. Some candidate vaccines rely on T cell epitopes present in a carrier, for instance vaccine CAD106 consists of the B cell epitope Aβ1–6 linked to the capsid of the Qβ bacteriophage, and vaccine ABvac40 consists of the B cell epitope Aβ33-40 conjugated to a carrier protein, keyhole limpet hemocyanine (KLH) (Table 1).

Other candidate vaccines consist of synthetic peptides that include T cell epitopes from pathogens: vaccine UB-311 is a mixture of two synthetic peptides, each including the B cell epitope Aβ1–14 and a T cell epitope either from the hepatitis virus or the measles virus; vaccine Lu AF20513 includes B cell epitope Aβ1–12 and T cell epitopes from tetanus toxin (Table 1).

None of the second-generation vaccines that entered clinical trials have induced meningoencephalitis, whereas antibody-mediated adverse effects, such as ARIA, were reported. No clinical benefit from the second-generation vaccines has been reported so far.

Although no direct comparison has been published, the duration of the anti-Aβ response elicited by second generation vaccines appears shorter than the duration of the response elicited by the AN1792; vaccine-induced antibodies become undetectable a few months after the last injection.

In the case of ACC-001 [72], substantial interindividual variability in anti-Aβ IgG titer was observed, and no correlation was found between IgG titer levels and cognitive or functional efficacy results, or biomarker results [72].

CAD106 induced an antibody response against Aβ in most patients. No meningoencephalitis was observed. CAD106-induced antibodies reacted with amyloid plaque cores and Aβ oligomers ex vivo. The best reactivity was seen with the entire Aβ1–6 epitope. No statistically significant differences were observed in CSF biomarkers [73].

## 8. Open Problems in Anti-Aβ Vaccine Development

The efficacy of vaccinations typically correlates with a threshold titer of the antibodies that mediate protection, and the duration of immunity following vaccination reflects the persistence of the antibody titer above the protecting threshold. One of the roadblocks in the clinical development of an efficacious anti-β amyloid vaccine has been the difficulty of obtaining a high titer, long-persisting antibody response to β-amyloid in all vaccinees; the threshold level of anti-Aβ antibodies predictive of vaccine efficacy has not been identified yet. 

In a preclinical study, we have observed that a candidate anti-Aβ vaccine, consisting of a filamentous phage fd displaying on its main capsid protein the epitope Aβ(2–6), was able to delay the onset of plaque deposition, only in some immunization schedules. Plaque pathology was prevented by a chronic immunization protocol involving monthly injections of vaccine, whereas an immunization protocol involving only two doses did not prevent plaque pathology, despite inducing a persistent anti-Aβ antibodies titer [74]. We hypothesized that the efficacy of the immunization protocol was related to the antibody titer achieved, and estimated the antibody titer required for the prevention of plaque pathology to be 1:10000 [74]. However, we subsequently observed that the ELISA-measured anti-Aβ titer is not sufficient to define the efficacy of the antibody response. A different candidate vaccine, (1–11)E2, consisting of a multimeric protein displaying the epitope Aβ(1–11), failed to prevent plaque pathology in mice, despite inducing anti-Aβ titers higher than 1:10,000 in many individuals. In mice immunized with (1–11)E2, we observed no difference in β-amyloid load between high responders, with antibody titers above 1:10,000 and low responders, with titers below 1:10,000, suggesting that the total anti Aβ titer is not a good correlate of efficacy, and that it is necessary to identify the qualitative features of the antibody response that correlate with efficacy [34,75]. In the case of vaccine (1–11)E2, antisera preferentially recognized aggregated Aβ species, namely oligomers, protofibrils and fibrils, however we observed a higher titer of antibodies recognizing higher-order Aβ aggregates, such as fibril and protofibrils, compared to anti-Aβ oligomer antibodies [36]. As previously discussed, such a pattern may reflect avidity effects, and does not imply the recognition of a conformational epitope. While the ability to bind aggregated Aβ species had previously been associated with the ability of an antibody to reduce plaque load [42], in the mice immunized with (1–11)E2 the antibodies had no effect on plaques, despite their ability to bind aggregate forms. It is possible that the prevalence of the IgG1 isotype in the antibody response might have limited the efficacy of this vaccination protocol. The identification of the features of a polyclonal anti-Aβ antibody response that correlate with the ability to interfere with the accumulation of β-amyloid, and Alzheimer’s disease, remains an open problem that deserves further investigation. The comparison of different vaccination protocols, in terms of quantitative and qualitative features of the immune response, will be instrumental to the identification of the correlates of efficacy. 

Both the titer and affinity of the antibody response can be modulated by the adjuvant used and by the immunization schedule. We have investigated, in silico and in vivo, the effect of the time interval between the first and the second dose on the magnitude of the antibody response to the N terminal epitope of β-amyloid, displayed on a filamentous bacteriophage or a multimeric protein.

We observed that some immunization schedules interfered with the development of immunological memory, resulting in a reduced response to the β-amyloid epitope in some individuals [76,77,78]. The immunization schedule is important to obtain high antibody titers in all vaccinees; as it is not feasible to test in humans a large number of different schedules, in silico simulations can be useful and a better understanding of the immunological mechanisms underlying these effects is desirable.

Another important roadblock in the development of a vaccine is the complexity of Alzheimer’s disease neuropathology, which is not fully captured by pre-clinical mouse models of β-amyloid deposition. In humans, cardiovascular disease, cerebrovascular disease, metabolic and psychiatric factors, diet, lifestyle, and education can modify the risk of developing dementia [79]. Elderly people with Alzheimer’s disease have an increased incidence of vascular brain injury, strokes, and microvascular infarcts [79]. Some patients have mixed forms of dementia, that include both the neuropathological hallmarks of Alzheimer’s, and cerebrovascular disease [80]. Less commonly, patients with Alzheimer’s disease may also have Lewy bodies, which are abnormal intracellular protein aggregates typical of Parkinson’s disease and dementia with Lewy bodies [79]. The first clinical trials of anti-β-amyloid vaccination did not use biomarkers to confirm AD diagnosis, so a non-negligible number of amyloid-negative patients, affected by other forms of dementia, was included, and this may have affected the results; current studies verify biomarkers in enrolled patients.

The complexity and interindividual diversity has several implications in the development of a vaccine for the prevention of Alzheimer’s disease. It is possible that a given vaccine may only be effective in a subset of people, therefore it is important to identify the homogeneous subgroups that can benefit from specific interventions. In addition, the prevention of Alzheimer’s disease and mixed forms of dementia may require combined approaches. One possibility is to target both Aβ and tau immunotherapeutically [81,82]. Treatment of hypertension, more childhood education, exercise, maintaining social engagement, reducing smoking, and management of hearing loss, depression, diabetes, and obesity are all considered promising strategies in the prevention of dementia [79]. Patients with vascular risk factors, cardiovascular disease and cerebrovascular disease are currently excluded from clinical studies of anti-Aβ vaccination. Once a successful immunotherapy is developed, it will be important to establish if these people can benefit, as they account for a large percentage of the subjects with dementia [80].

## 9. Passive Anti-Aβ Immunization 

In parallel with the evolution of anti-Aβ vaccination strategies, many passive anti-Aβ immunotherapies have been attempted. Passive immunotherapy consists in the administration of antibodies, and thus circumvent some problems that hampered active immunotherapy, such as the T cell mediated adverse reactions, and the interindividual variability in the antibody titer and in the specificity of the antibody response. On the other hand, while the natural antibody response is polyclonal and involves different isotypes, treatment with a monoclonal antibody may rely on a more limited set of mechanisms of action. Passive anti-Aβ immunotherapy has been reviewed in detail elsewhere [5,82], and an extensive discussion of this approach is beyond the scope of this review. However, in the context of the development of a vaccine, the clinical results of passive immunotherapy with anti-Aβ monoclonal antibodies provide invaluable information about the effects of the titer, isotype, and epitope specificity of antibodies, and the importance of the timing of intervention. Four monoclonal anti-β-amyloid antibodies are currently in phase III of clinical testing: Aducanumab, BAN2401, Gantenerumab, and Solanezumab (Table 2). 

The monoclonal anti-β-amyloid antibody that is currently in a more advanced stage of clinical development is Aducanumab, a human IgG1 antibody obtained from a screening of memory B cells from healthy aged people, which selectively targets aggregated forms of Aβ, including soluble oligomers and insoluble fibrils [30]. In a phase Ib trial, the higher doses of aducanumab tested (3 and 10 mg) reduced PET amyloid levels at six months and more so at 12 months, when the highest dose reduced cortical amyloid close to the cut point of positivity [30]. This dose-dependent evidence of target engagement and biomarker movement was accompanied by significantly less clinical decline compared to placebo in two tests, the Mini-Mental State Exam and the Clinical Dementia Rating—Sum of Boxes [30]. Transient brain edema was observed in around 20% of patients, mostly ApoE4 carriers, was dose-dependent, but produced no symptoms in 65% of cases and was resolved in all cases; therefore, the safety and tolerability were considered acceptable [30]. Aducanumab was then tested in phase II trials ENGAGE (NCT02477800) and EMERGE (NCT02484547) in people with mild cognitive impairment due to AD or mild AD as ascertained by a positive amyloid PET scan; trial participants received monthly infusions of one of three doses of aducanumab or placebo for 18-months. The two trials were discontinued following a futility analysis, but Biogen subsequently announced that additional data analysis indicated that longer exposure to the higher dose might be effective. The trials’ results have not been published yet, and doubts have been expressed about the validity and clinical significance of the results [83].

Gantenerumab, an IgG1 antibody, was selected from a fully synthetic phage library containing functional human antibody genes [84]; the library contained antibody frameworks based on the combination of the most used heavy and light chain variable region genes, in which synthetic CDR3 cassettes were inserted [85]. Gantenerumab was optimized in vitro for binding with sub-nanomolar affinity to a conformational epitope expressed on β-amyloid fibrils [86]; it binds to both N-terminal and central regions of Aβ, which are exposed in close juxtaposition at the surface of fibrils [87]. The X-ray structure of a complex of the Fab of gantenerumab to Aβ1–11 revealed an orientation of the N-terminal Aβ segment in the antigen-binding cleft opposite to the orientation described for other Fab-Aβ complexes and it displays a comparable affinity for oligomers and fibrils, and about 10× lower affinity for monomers [86]. Gantenerumab did not show clinical benefit in familial AD subjects, however, it revealed a lowering of amyloid PET, accompanied by an important decrease in CSF ptau and brain tau-PET [88].

Solanezumab, a humanized IgG1 analog of a murine antibody that targets the central domain of Aβ and is selective for soluble forms, failed to improve cognition or functional ability in patients with mild-to-moderate Alzheimer’s disease [89], and in patients with mild dementia due to Alzheimer’s disease [41], and did not significantly slow down the rate of brain atrophy. It is currently being tested on asymptomatic or very mildly symptomatic people 65 and older who have biomarker evidence of brain amyloid deposition [90]. 

BAN2401 is a humanized, IgG1 version of a mouse monoclonal antibody, which selectively binds soluble Aβ protofibrils. It was safe and well tolerated in mild to moderate Alzheimer’s disease [91]. Treatment with BAN2401 was associated with a reduction for biomarkers in the cerebrospinal fluid (p-tau, neurogranin), more pronounced for ApoE4 carriers [92]. According to results from a phase II trial presented at the Alzheimer’s Association International Conference 2018, and not published yet, treatment with BAN2401, at the highest dose, over 18 months, reduced amyloid in early Alzheimer’s disease and slowed cognitive decline.

Different binding specificity to the different Aβ species characterize the different anti-Aβ monoclonals [93]. While solanezumab disrupts the initial formation of fibrils, gantenerumab slows their elongation. Aducanumab coats the surface of fibrils, with the result that fibrils cannot interact with monomers and fewer oligomers are generated [93]. 

## 10. Perspectives

Clinical trials have shown that immunization against β-amyloid can reduce amyloid load and that β-amyloid removal has clinical effects on memory and activities of daily living, but the magnitude of these effects has been disappointing so far. There is evidence that the benefits of vaccination accrue over time, however in the AN1792 trial most patients progressed to severe dementia, including those with very extensive plaque removal. Therefore, it is clear that, at some stages of pathogenesis, plaque removal does not stop the progression of the disease, possibly due to continued tau propagation. 

The current working hypothesis, and hope, is that there is a pre-symptomatic stage in the pathogenesis when the removal of amyloid can significantly delay the onset of clinical disease. Failure of anti-Aβ immunotherapies is often attributed to an insufficient titer or inappropriate timing of the response (“too little, too late”) [75]. Also, it is clear that the specificity of antibodies against different Aβ species affects the outcome. Future vaccination attempts need to address the earliest stages of the pathogenesis, and optimize the titer of the antibody response. Moreover, the response should be focused against the most pathogenic Aβ species. 

It is still not clear how early in the pathogenic process the vaccination needs to be performed. Even the preclinical, asymptomatic phase of Alzheimer’s occurs after a much earlier pathogenic process, the formation of small oligomeric seeds of misfolded Aβ. The highly bioactive Aβ seeds are, in perspective, an extremely interesting targets for a truly prophylactic vaccination protocol. It would be very important to try and develop immunogens that mimic epitopes of the Aβ seed. 

The characterization of the Aβ species recognized by antibodies is essential to vaccine development. Recently, a technique termed antibody recognition profiling of Aβ assemblies (ARPA) has been described, that consists in the separation of brain-derived Aβ assemblies by agarose gel electrophoresis, followed by enzymatic digestion of the agarose to liberate the Aβ assemblies, and antibody immunoprecipitation (IP) to establish the recognition profile for a given antibody against different Aβ species. This assay has been instrumental in characterizing the specificity of the antibody Aducanumab, which appears able to neutralize Aβ seeds [23]. Ideally in vaccine development antisera should be characterized with a set of standard assays, to allow meaningful comparisons.

The complex pathophysiology of AD may require combination treatments, for instance addressing tau protein and cerebrovascular disease at the same time. Moreover, due to interindividual differences, it is possible that only particular subsets of patients may benefit from a given anti-Aβ immunotherapy, therefore it is important to subtype patients according to APOE genotype, risk factors, immune phenotype, clinical symptoms, cerebrovascular parameters, biomarkers, neuroimaging, and Aβ strain. 

Passive anti-Aβ immunotherapy at the moment appears closer to success than active vaccination, because the dose and specificity of the antibody are more controllable. An advantage of vaccination is that in general its effects are more long-term that the effects of monoclonal antibodies, and moreover a polyclonal response that includes different specificities and isotypes may be advantageous. However, even an acute treatment with monoclonal antibodies could in principle have an efficacy much longer than the half-life of the antibody, if the treatment “turned back the clock” by years in the pathogenic process. At the moment, it seems important that both active and passive immunotherapy strategies are further investigated. 

## 11. Conclusions

After two decades of research in the field, the prevention of Alzheimer’s disease by vaccination against β-amyloid appears a very difficult objective to achieve, but not an impossible one. Clinical trials of vaccinations and passive immunotherapy have shown that antibodies can slowly reduce the amyloid load in the brain. Effects on cognition have been reported, although so far the effects are disappointingly small. Nevertheless, the directions to explore in future attempts are clear. Intervening as early as possible in the pathogenesis, achieving higher antibody titers, and optimizing the qualitative features of the antibody response could result in a vaccine that, combined with other interventions, may reduce the incidence of Alzheimer’s disease. Further research is warranted.

## Figures and Tables

**Table 1:** Vaccines against β-amyloid that entered phase II clinical trials.

	Epitope	Composition	Safety Immunogenicity Clinical Effects
AN1792	Aβ42	Pre-aggregatedAβ42 with QS-21 adjuvant	Unsafe, immunogenic, some clinical effect
AD02	Mimics Aβ N-terminus	6-mer peptide with alum adjuvant	Safe, immunogenic, no clinical effect
CAD106	Aβ1–6	Aβ1–6 coupled to a Qβ phage capsid	Safe, immunogenic, no clinical effect
ACC-001	Aβ 1–7	Aβ1–7 coupled to inactivated diphtheria toxin with QS-21 adjuvant	Safe, immunogenic, no clinical effect
ACI-24	Aβ1–15	Aβ1–15 anchored by both ends into the surface of liposomes	Poorly immunogenic
Lu AF20513	Aβ1–12	Three repeats of Aβ1–12 interspersed with T epitopes of tetanus toxin	No published data
UB-311	Aβ1–14	A mixture of two peptides, each comprising Aβ1–14 and a T epitope with alum and CpG	Safe, immunogenic, no published efficacy data
ABvac40	Aβ33–40	Aβ33–40 conjugated to KLH, with alum	Safe, immunogenic, phase II trial to end in 2022

**Table 2:** Monoclonal antibodies against β-amyloid currently in phase III clinical trials

	Specificity	Mechanism of Action	Clinical Effects
Aducanumab	Aggregated Aβ	Reduces fibril-dependent formation of oligomers	Long exposure to high dose might reduce cognitive decline (unpublished results)
BAN2401	Soluble Aβ protofibrils	Reduces Aβ protofibril toxicity	Long exposure to high dose might reduce cognitive decline (unpublished results)
Gantenerumab	Aβ oligomers and fibrils	Slows fibril elongation	No clinical benefit reported so far. Lowers amyloid PET, tau-PET and CSF ptau
Solanezumab	Soluble Aβ	“Peripheral sink” effect was expected. Data refute the peripheral sink hypothesis.	No clinical benefit reported so far. Currently tested on asymptomatic or very mildly symptomatic people 65 and older who have biomarker evidence of brain amyloid deposition
